# “Early intervention isn't an option, it's a necessity”: learning from implementation facilitators and challenges from the rapid scaling of an early intervention eating disorders programme in England

**DOI:** 10.3389/frhs.2023.1253966

**Published:** 2024-01-18

**Authors:** Lucy Hyam, Claire Torkelson, Katie Richards, Amy Semple, Karina L. Allen, Jill Owens, Aileen Jackson, Laura Semple, Danielle Glennon, Giulia Di Clemente, Ulrike Schmidt

**Affiliations:** ^1^ Centre for Research in Eating and Weight Disorders, Department of Psychological Medicine, King’s College London, Institute of Psychiatry, Psychology and Neuroscience, London, United Kingdom; ^2^ Department of Psychosis Studies, King’s College London, Institute of Psychiatry, Psychology and Neuroscience, London, United Kingdom; ^3^ Centre for Implementation Science, King’s College London, Health Service and Population Research Department, Institute of Psychiatry, Psychology and Neuroscience, London, United Kingdom; ^4^ Health Innovation Network, Academic Health Science Network South London, London, United Kingdom; ^5^ South London and Maudsley NHS Foundation Trust, London, United Kingdom; ^6^ Eating Disorders Outpatients Service, Maudsley Hospital, South London and Maudsley NHS Foundation Trust, London, United Kingdom

**Keywords:** feeding and eating disorders, National Health Services, early medical intervention, mental health services, emerging adulthood, implementation research

## Abstract

**Introduction:**

The First Episode Rapid Early Intervention for Eating Disorders (FREED) service has shown promising outcomes for young people with an eating disorder, leading to national scaling and implementation across England. Between 2020 and 2023, the national implementation of FREED was supported by the Academic Health Science Networks (AHSNs), which are publicly funded organisations with the mission to spread innovations at scale and pace. This study aimed to investigate the views and experiences of AHSN programme leads on the national roll-out of FREED and the perceived sustainability of the model.

**Methods and results:**

Semi-structured interviews were conducted with 13 programme leads across the AHSNs with direct experience supporting the national implementation of FREED. Thematic analysis was adopted using a critical realist approach. Initial sub-themes were inductively generated and then organised under seven larger themes representing the domains of the Non-adoption, Abandonment, and Challenges to Scale-Up, Spread and Sustainability (NASSS) framework. Each sub-theme was classified as a facilitator and/or barrier and then each larger theme/domain was assessed for its complexity (simple, complicated, complex). Data analysis revealed 28 sub-themes, 10 identified as facilitators, 13 as barriers, and five as both. Two domains were classed as simple, three as complicated, and two as complex. Sub-themes ranged from illness-related complexities to organisational pressures. Key facilitators included a high-value proposition for FREED and a supportive network. Key barriers included staffing issues and illness-related factors that challenge early intervention.

**Discussion:**

Participants described broad support for FREED but desired sustained investment for continued provision and improving implementation fidelity. Future development areas raised by participants included enlarging the evidence base for early intervention, increasing associated training opportunities, and widening the reach of FREED. Results offer learning for early intervention in eating disorders and the scaling of new health initiatives.

## Introduction

Early intervention efforts in psychiatry emphasise the detection of a disorder at the earliest possible stage, followed by interventions proportional to the stage of illness (1). In psychosis, where the evidence is most established, early intervention has globally transformed the understanding of psychosis and has influenced service reform (2). Whilst research on early intervention for eating disorders (EDs) is still in its infancy, the First Episode Rapid Early Intervention for Eating Disorders (FREED) pathway has shown promising outcomes for patients in the United Kingdom (3–5). The case for early intervention for EDs stems from neurobiological, clinical, and socioeconomic evidence that early intervention may improve outcomes and sustain full recovery from an ED (6, 7).

However, individuals often do not receive treatment for an ED until many years after first experiencing symptoms (8). There are many barriers to accessing treatment for an ED, including important patient factors such as delays in help-seeking (9, 10). There are also widescale service-related restrictions to accessing care in the United Kingdom. In some areas, only low-weight patients are prioritised for treatment, with higher-weight patients being regarded as “not sick enough” to access treatment (11), although recent guidance on early intervention clearly states that this is not acceptable practice (12). Early intervention for EDs has recently been rated as a priority for investment in the United Kingdom, with best practice recommendations advocating for sufficient funding for frontline services and early intervention initiatives (13).

FREED is one approach to reducing service-related delays to specialist ED treatment. FREED is a trans-diagnostic service model and care package for young people aged 16–25 years with a recent onset ED that is 3 years or less in duration (14). FREED encourages rapid access to treatment that is well-coordinated, evidence-based, and adapted to young people's needs (9, 11). It operates as a “service within a service.” Within each site, a FREED Champion is appointed to manage the provision of the service and coordinate a “mini team” of clinicians who deliver treatments adapted to the FREED model and ethos. Early access to evidence-based treatment is encouraged through wait-time targets of 2 weeks for an assessment to take place and 4 weeks for starting treatment concordant with national [National Institute for Health and Care Excellence (NICE)] guidelines.

**Figure 1:**
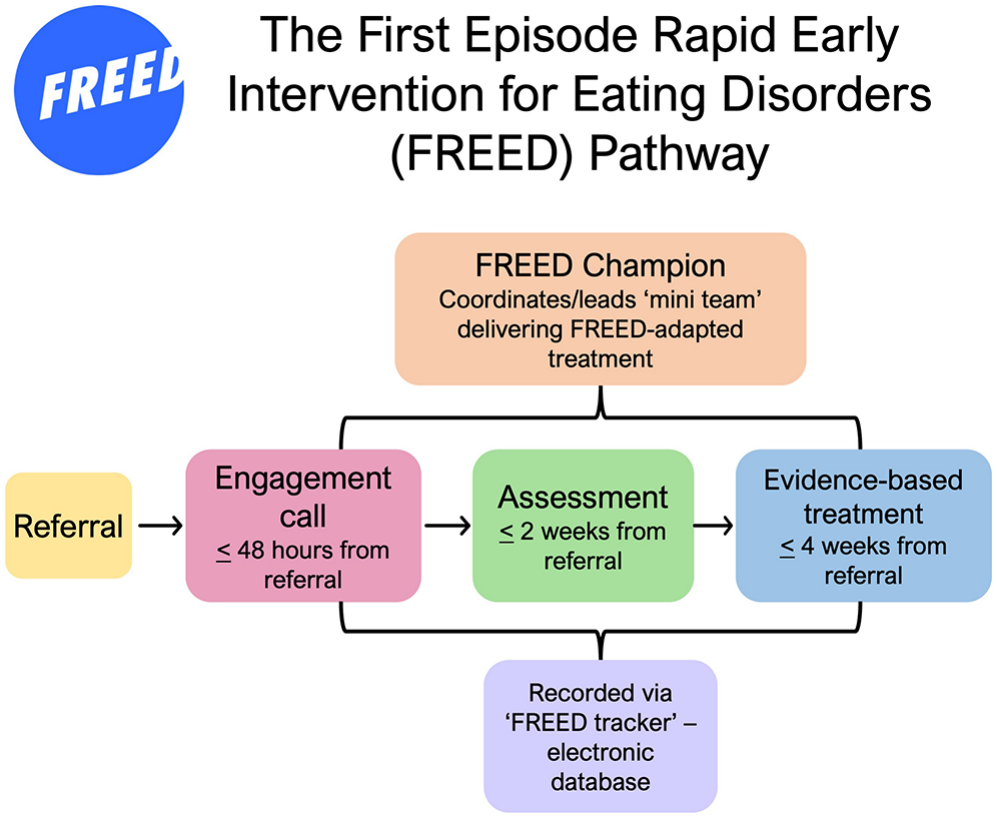
The First Episode Rapid Early Intervention for Eating Disorders (FREED) pathway.

Active outreach to patients is encouraged via a phone call within 48 h of referral to praise help-seeking and screen eligibility for the service. All services using FREED become part of the FREED network, overseen by a core national team. This national team provides training, implementation support, and ongoing evaluation to the growing FREED network and comprises clinicians and academics from the South London and Maudsley NHS Foundation Trust (SLaM) and King's College London (KCL) (9, 15). Online training is freely available via the FREED website (freedfromed.co.uk), and live training supplements this. Due to growing treatment waiting lists, a more recent addition to FREED involves recording other forms of support/intervention offered to patients that do not come under NICE guidelines for treatment, for example, psychoeducational groups or peer support sessions. The FREED pathway and processes are depicted in Figure 1.

The FREED model has been evaluated in a single-centre pilot study and a multi-site (FREED-Upscaled) study, demonstrating reductions in the duration of untreated eating disorder (DUED), significant improvements in clinical outcomes, and notable cost savings (3, 4, 11, 14). Patients treated through FREED valued timely access to treatment and developmentally tailored adaptations to treatment (16).

**Figure 2:**
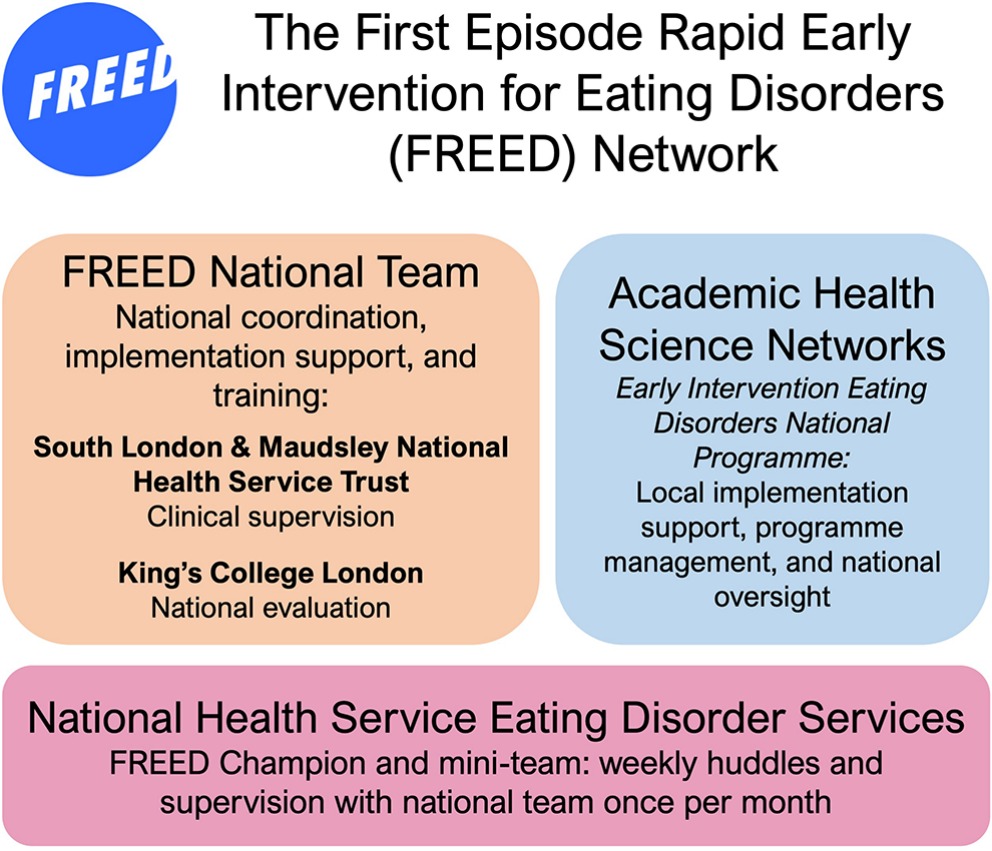
The First Episode Rapid Early Intervention for Eating Disorders (FREED) network.

Since these earlier evaluations, significant investment has been provided by the National Health Service (NHS) and Academic Health Science Networks (AHSNs) to scale FREED nationally (6). AHSNs were established by NHS England to address national and local healthcare priorities by improving the spread of innovation and improvement, with regional implementation specialists managing the adoption of initiatives within their local area (17). In April 2020, FREED became part of the AHSNs' national adoption and spread programme. This supported its scaling across England, with 15 localised “Early Intervention Eating Disorder” programme leads managing the implementation of FREED in local ED services (5, 18). By the end of the AHSN national programme in March 2023, 46 NHS trusts had adopted or started implementing FREED out of 54 eligible NHS Trusts across England. A summary of the national roll-out is depicted in Figure 2.

Data from the evaluation of this national scaling (the “FREED-4-All” dataset) suggest that findings from the earlier studies were replicated here (5). Most of this scaling took place after the onset of the COVID-19 pandemic when ED services internationally faced challenging conditions including increasing numbers of referrals (19). Even before the onset of the pandemic, UK adult community ED services experienced a growing demand and were underfunded/understaffed (20). There is currently no qualitative exploration of the experiences of those implementing FREED during this time. This is important if the challenges and facilitators associated with implementing FREED are to be understood, to inform future planning for the delivery of authentic early intervention, and to ensure sustainability of the FREED model. Qualitative evidence from those directly involved in the implementation of FREED will provide a rich understanding of these facilitators and challenges, and will also be useful for other services looking to adopt an early intervention service.

The scaling, implementation, and evaluation of FREED to date have been guided by the RE-AIM implementation framework (Reach, Effectiveness/Efficacy, Adoption, Implementation, Maintenance; www.RE-AIM.org) (9, 15). Another implementation framework, which is well-suited for evaluating the next stage of scaling and implementation of FREED, is the Non-adoption, Abandonment, and Challenges to Scale-Up, Spread and Sustainability (NASSS) framework (21). The NASSS framework seeks to map areas of possible complexity in innovation projects by considering seven domains where complexity may arise and how these may lead to the abandonment or non-adoption of healthcare technology/innovations (22). This framework is suited for the next stage of evaluating the scaling and implementation as we look for ways to reduce and manage areas of complexity, maintain/sustain FREED, and improve local implementation fidelity.

The seven domains of the NASSS framework comprise the health condition(s) that FREED addresses (EDs); the technology/service (FREED) and its functionality; the value proposition of FREED to the whole healthcare system; the useability and acceptability of FREED for adopters (NHS ED service staff); organisational (NHS ED services) factors; wider context issues (e.g., policy-related drivers); and an overarching consideration of the emergence and adaptation of FREED over time. Considering ways to reduce innovation complexity within real-world and “rough ground” implementation of innovations is vital for futureproofing an intervention, and this rough-ground implementation is where attention in innovation projects should be directed (23).

The current study therefore seeks to use the NASSS framework to investigate the views and experiences of AHSN Early Intervention Eating Disorder (EIED) programme leads in supporting ED services to implement FREED, as well as to seek their views and opinions on the sustainability of FREED. The study aims are to understand (1) the perceived challenges and facilitators to implementing FREED in ED services across England and adaptations made to the model during implementation and (2) explore views on the sustainability of FREED.

## Materials and methods

An exploratory, qualitative study design involving individual semi-structured interviews was used for this study. A critical realist perspective was adopted, which assumes the existence of an objective world that can be known through scientific endeavour, but our understanding is gained through subjective human experiences and interpretation (24). Critical realism emphasises a theory-based (not determined) approach to gaining knowledge; it posits that our understanding of reality is mediated through theoretical frameworks and interpretations of researchers (25). As such, we seek to understand the unique and subjective experience of those involved in implementing FREED as guided by the NASSS framework. Ethical approval for this study was granted by King's College London Minimal Risk Ethics (MRA-21/22-26307).

### Procedure

We invited all 15 AHSN early intervention ED national programme leads to participate in this research via email invitation and verbal invitation at network meetings. After a review of a participant information sheet, written informed consent was sought from participants. A copy of the interview schedule and an outline of the NASSS framework were sent to the participants before the interview so they could familiarise themselves with the framework and questions ahead of time. The interview schedule was constructed with the aid of the NASSS-Complexity Assessment Tool, which is a toolkit designed to help research and evaluate health change projects (26). Twenty-seven questions were divided into three sections: (1) Getting FREED started, to consider the early stages of engaging services/stakeholders; (2) Implementing and embedding FREED, to consider implementation challenges and facilitators pre- and post-launch; and (3) sustaining and adapting FREED, to consider issues regarding sustainability and suggestions of how FREED can be developed.

### Data collection

Data collection took place between March and June 2022. All interviews took place virtually via Microsoft Teams and lasted for an average of 46 min (range 26–57 min). Either one or two researchers (LH and CT) were present for the interviews. The interviews were transcribed using Microsoft Stream transcription software and checked for accuracy by CT against the original video recording.

The researchers involved in the study carefully reflected on possible sources of bias throughout the design of the study and when collecting and analysing data. Researcher CT was independent of the FREED team, whereas researcher LH was part of the separate evaluation team within the FREED network. The interview schedule was carefully structured to try and ensure a balanced exploration of both positive and negative views. Throughout the interview, it was reiterated that confidentiality would be held to any views expressed. The interview schedule was used flexibly to ensure that participants' lines of reflection and commentary were pursued.

### Analysis

Data analysis was supported by NVivo 12 (27). Transcripts were read multiple times for data immersion. Reflexive thematic analysis was used for the interviews in an iterative, cycled process using inductive and deductive approaches. In the first cycle of analysis, six transcripts were independently coded by two authors (LH and CT) with inductive codes that were relevant to the research questions. While the first cycle of coding was inductive/data-driven, the research aims and interview schedule were informed by the NASSS framework. The framework would therefore have influenced the data and initial inductive/data-driven coding.

The initial codes were reviewed for triangulation and then mapped into a coding framework by LH and CT. Codes were organised into sub-themes and organised under the seven broader theme domains of the NASSS framework to identify distinct areas where complexity may have arisen in the implementation of FREED. In the second cycle of analysis, LH and CT coded the remaining seven transcripts according to the coding framework, with researchers allowing for the development of any new codes arising from the data.

This process was continually refined in an iterative process, with modifications being made to the coding framework to capture new arising concepts. The first six transcripts were also checked again to see if new codes could be applied to these transcripts. Any codes or sub-themes that did not fit with the overall analysis were first checked to see if they could be merged with another code or sub-theme. If they were not well-supported or could not be merged with other codes, they were deleted (28). The final sub-themes that emerged were then classified under the domains as either a facilitator and/or barrier. The final stage of analysis involved classifying the complexity of each domain as either simple (predictable, few components), complicated (many components, predictable), or complex (many components interacting unpredictably) (21) by LH with a review from author US. This was determined by reviewing the sub-themes under each domain and based on a theoretical understanding of the NASSS framework. A similar approach using the NASSS framework has previously been taken elsewhere (29).

## Results

Thirteen participants representing 12 of 15 different AHSN regions and the national AHSN programme were included in the sample. Each participant supported multiple ED services to adopt FREED, and most had experience managing other mental healthcare projects alongside a portfolio of physical health improvement projects. All participants had an active role in facilitating the adoption and implementation of FREED, for example, by managing regional funding, engaging with relevant stakeholders, sourcing implementation materials for teams, and facilitating shared learning opportunities.

**Figure 3:**
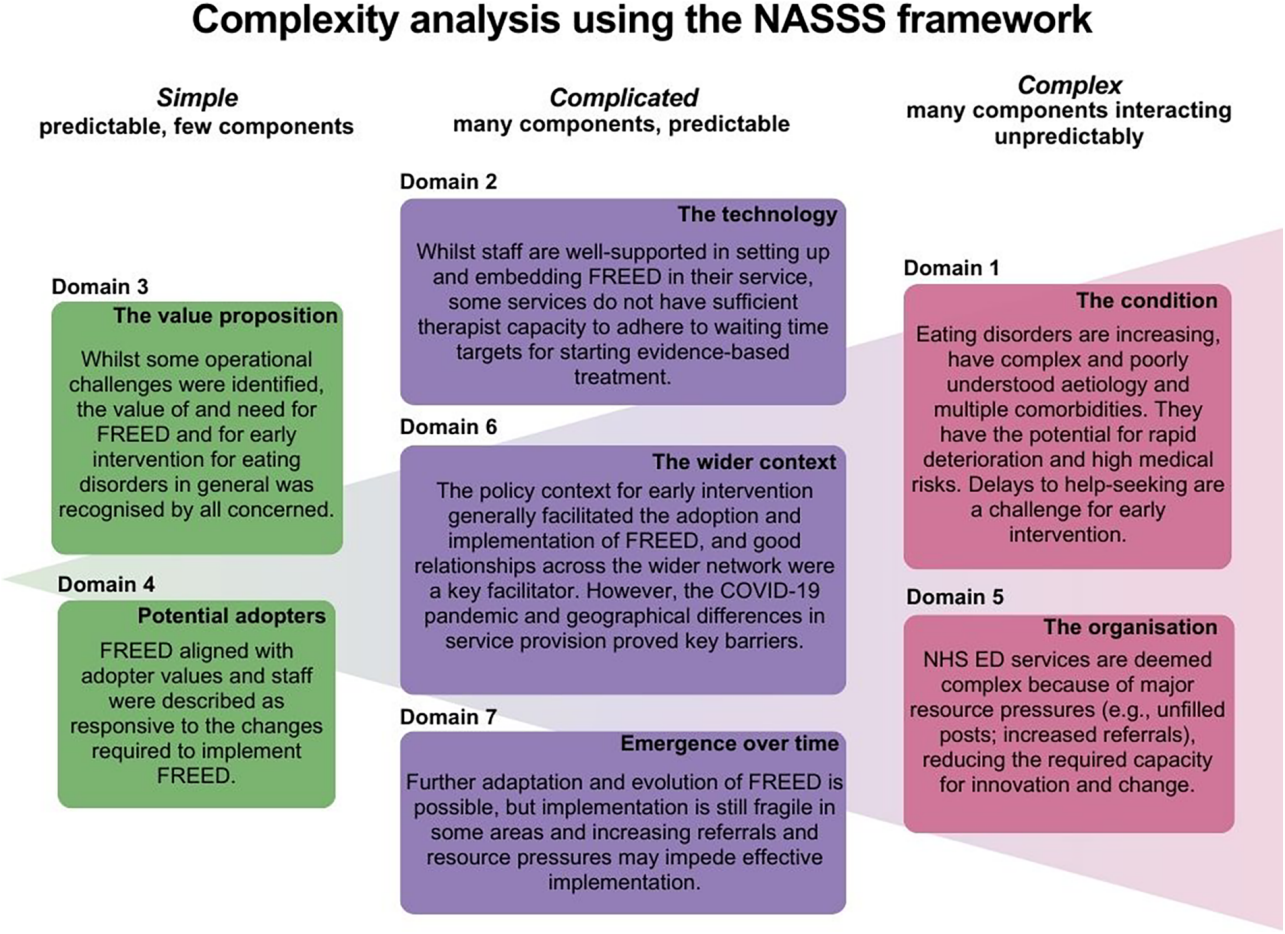
Complexity analysis of themes and sub-themes using the Non-adoption, Abandonment, and Challenges to Scale-Up, Spread and Sustainability (NASSS) framework.

Table 1 shows all themes and sub-themes and whether they were facilitators, barriers, or both. Data analysis revealed 28 sub-themes (10 facilitators, 13 barriers, and five considered as both). Two domains were classed as simple, three were classed as complicated, and two were classed as complex (Figure 3). Descriptions of the sub-themes felt to be most prominent are included in this manuscript. The additional sub-themes are described in Supplementary Data S1.

**Table 1:** Summary of NASSS domains with corresponding themes and sub-themes.

Domain and complexity rating	Sub-theme	Example data	Description	Facilitator/barrier
The condition (eating disorders) *Complex*	Understanding of eating disorders	“Eating disorders are* … *not well looked after, not well funded, and in general not well or not fully researched.” P5	• Poor societal understanding of eating disorders • Participants had good background knowledge, which fuelled enthusiasm	Facilitator and barrier
	Illness complexity	“With people who are referred to eating disorder services* … *the level of acuteness had gone sky high.” P4	• Delays to help-seeking challenge early intervention • Increasing acuity of cases	Barrier
The technology (FREED) *Complicated*	Readily available and stable investment	“Help them secure sustainable funding. That's been absolutely critical.*”* P2	• Funding opportunities were not equitable • Easy to mobilise when the investment was readily available or awarded	Facilitator and barrier
	KCL/SLaM national team resources and support	“I think the documentation has been really clear for this as well…” P12	• FREED national team and materials were supportive and significant facilitators	Facilitator
	Accessing training	“We absolutely need more people to be able to deliver MANTRA training.” P9	• Online training was helpful, but there was a desire for live training • More training on how to deliver evidence-based treatments was desired	Barrier
	Adhering to waiting time targets	“It's very resource intensive* … *hitting the 48 h, 2 weeks, 4 weeks…” P7	• Adhering to FREED wait time targets is variable across sites, but was perceived as generally attainable	Facilitator
The value proposition *Simple*	High desirability and value	“Everyone I speak to understands* … *the drive and the need for this* … *which is not always the case, I'll add, in some national programs.” P9	• Participants were excited about FREED, but this was frequently paired with pessimism about operational challenges on the ground	Facilitator
	Patients outside of the eligibility criteria	“So I think, whilst FREED's great for 16–year-olds* … *let's think about the over 25 s.” P7	• There was a desire for early intervention for patients over 25 years or with a longer duration of illness	Barrier
	Capturing the wider benefits of FREED	“I just didn't want people to think that we were failing as a programme because we weren't meeting those treatment target times.” P1	• There was difficulty demonstrating the wider benefits of FREED in addition to the identified outputs of the AHSN national programme	Barrier
	FREED's evidence base	“I think the evidence base has been absolutely critical.” P2	• FREED evidence base was described as critical to adoption	Facilitator
	Buy-in required across multiple systems	“Getting that buy-in across complex systems. That's a real challenge to getting started.” P3	• AHSN leads needed to gain buy-in across multiple systems	Barrier
Potential adopters (NHS ED clinicians) of the technology *Simple*	Support needed from AHSN	“It's something that they might not have had the time or headspace to do themselves.” P5	• Support provided by participants was described as a key facilitator to getting FREED started and implemented	Facilitator
	Engaged and passionate clinicians	“I think they were excited about this kind of innovation and have been really responsive to things.” P12	• Enthusiasm of clinicians and FREED Champions who truly embodied “champion” role in teams	Facilitator
The organisation (NHS eating disorder services) *Complex*	Conditions of implementation	“We offer FREED to 18–19-year-olds rather than the full age group, and only to mild and moderate presentations.” P3	• Variable implementation fidelity • Rapid vs. slow implementation • One-person vs. team approach	Barrier
	Balancing risks of and appetite for innovation	“Uhm so I think that's been a major challenge is some risk aversion. But it has been adjusted for in a sense by ensuring that the trust can go step-by-step.” P5	• Risk aversion sometimes affected implementation fidelity (e.g., only rolling out to certain diagnoses)	Barrier
	Leadership and internal relations of the organisation	“Organizational support, essential service wide support, were really critical for that initial stage.” P3	• Engagement from a wider team and support of leadership were perceived as critical	Facilitator and barrier
	Fit between ED services and FREED	“Having an all-age eating disorder pathway is a real benefit.” P2	• FREED was described as fitting well in general with eating disorder services	Facilitator and barrier
	Limited capacity of ED services	“I think the problem is that services are so stretched* … *early intervention is meant to* … *speed things up and it's not” P11	• Teams stretched to capacity, impacting the ability to deliver early intervention and meet targets	Barrier
	Staff recruitment and retention	“They've been through several recruitment processes and not been able to appoint.” P8	• Participants discussed significant difficulties in recruiting staff and high turnover rates within the NHS • Exacerbated by the COVID-19 pandemic	Barrier
The wider context *Complicated*	Relationships between AHSN, adopters and KCL/SLaM FREED national team	“We couldn't have done it without either side of us working so closely together.” P1	• Relationship between services and AHSNs was built on trust and support • AHSNs appreciated the expertise and responsiveness of the SLaM/KCL national team	Facilitator
	AHSNs bringing everyone together	“I think we do, our sites are quite good at engaging, but they're not necessarily* … *very proactive.” P6	• AHSNs had a specific role in bringing services together and with external networks, creating avenues for communication and collaboration	Facilitator
	COVID-19 disruption	“The problem is* … *that the pandemic meant that we have been* … *firefighting for the past two years.” P10	• COVID-19 placed focus on high-risk cases or business as usual rather than innovation	Barrier
	Geographical differences in population and service provision	“I* … *had some concerns about the ability for it to be implemented in the same manner it was designed from in London.” P9	• Navigating differences in population and service offers across the country	Barrier
	Policy context for early intervention	“It was it aligned to the current thinking, the long-term plan.” P1	• Early intervention was stated as a priority and supported by policy context • There were some issues engaging certain providers	Facilitator and barrier
	Good engagement with patient and carer groups	“So the relationship between our clinical lead and the patient and carer collective has been very beneficial.” P9	• Good connections with patient and carer collaboratives and university groups can foster wider buy-in	Facilitator
Emergence over time *Complicated*	Implementation and network stability	“I know obviously our services have their FREED supervision and I suppose the question is how do they get that forever* … *how long does that go on for?” P3	• AHSNs were positive about sustainability, given the right investment • Implementation in some areas was considered to still be fragile • Networking opportunities needed to be sustained	Barrier
	Increasing referrals and condition complexity	“Anecdotally there are more complex patients, as well, that people have seen come through.” P12	• Anecdotal evidence of increasing acuity and complexity of ED referrals	Barrier
	Need for flexibility and creativity	“Perhaps* … *adding more options to treatments* … *allowing more flexibility within the FREED model as to what's delivered.” P5	• Considerations of changes/flexibility in treatment avenues, Champion post, data requirements, and waiting time targets	Facilitator

FREED, First Episode Rapid Early Intervention for Eating Disorders; KCL, King's College London; SLaM, South London and Maudsley NHS Foundation Trust; MANTRA, Maudsley Model of Anorexia Nervosa Treatment for Adults; AHSN, Academic Health Sciences Network; NHS, National Health Service; ED, eating disorder.

### Theme 1/NASSS domain 1: the condition

Complexity rating: The condition domain was rated as complex due to prominent challenges treating EDs and concerns about increasing acuity of cases coming through to ED services. Two sub-themes emerged here.

#### Sub-theme “illness complexity”

Many participants discussed the general complexity of the condition, for example, issues that impact help-seeking such as stigma. Participants also recalled conversations with services about an overall increase in the acuity of ED cases, which they were worried might adversely affect the capacity to implement FREED effectively.

…*it's like with … a lot of serious mental health issues, there's a lot of taboo around it. There's a general reluctance because of the nature of the condition to actually seek and access help. Often, parents, teachers, friends, contemporaries struggle to encourage young people to seek help, because it's often hidden.* (P10)

A second sub-theme is provided in Supplementary Data S1.

### Theme 2/NASSS domain 2: the technology (FREED)

Complexity rating: The technology domain was rated as complicated, as whilst the support and materials provided by the national team were highly complimented, there were key concerns about funding stability. Four sub-themes emerged under this domain.

#### Sub-theme: “KCL/SLaM national team resources and support”

Participants described the implementation materials provided by the national team, such as business cases, as being “really clear” and “helpful” (P12). However, there was also a desire for more specific resources on launching FREED and a specific FREED network contact directory.

Participants praised the monthly FREED implementation supervision sessions provided by the national team as working “really well” (P1). The accessibility of these sessions was also cited as a facilitator to implementation and the availability and expertise of the national team was highlighted as an important facilitator to getting FREED started.

*And the SLaM team have always been really, really open and responsive, which has been great and I think that's really helped to get things off the ground sooner.* (P5)

#### Sub-theme “accessing training”

Participants described difficulties for teams in accessing FREED training days. Whilst the online training package was described as helpful, there was a desire for more regular live training sessions for all new staff; participants wished for “provision for non-new sites, that have new staff joining, to have training available … on a regular basis…” (P7).

Many participants desired more training for clinicians to be able to deliver evidence-based treatments. For example, participants felt that training in the Maudsley Model of Anorexia Nervosa Treatment for Adults (MANTRA, one of three NICE-recommended treatments for adults with anorexia nervosa), would “allow teams to build their skill set, to be able to develop the skills to be able to deliver the treatments” (P10). Not having access to this training was perceived to impede efforts in managing increased referral numbers/need for treatment.

#### Sub-theme “readily available and stable investment”

A key perceived facilitator was having readily available funding for FREED, such as the NHS England pump priming that had been made available for 18 ED services (30). This was often described as the main catalyst for getting FREED started, even when the investment was modest. Being able to approach clinical leads and managers with funding for FREED demonstrated that the AHSNs had “skin in the game” (P10). Participants also described their support as instrumental in helping ED services to bid for and access funding opportunities.

*It literally was the money that opened the door. That £35,000 said “we are serious and … you can use this to put towards a FREED Champion.’ I think that was a game changer.* (P10)

Whilst funding opportunities were available for some sites and some services were successful in their bid for NHS funding, others were not, and this presented a direct obstacle to implementing FREED. Where funding was not readily available, participants became “frustrated” (P7) and wondered, “how can we ask people to do this when there's no money attached to it?” (P1). When thinking about the future of FREED, some participants were “pessimistic” (P11) about the potential instability of funding to source FREED.

A fourth sub-theme can be found in Supplementary Material S1.

### Theme 3/NASSS domain 3: the value proposition

Complexity rating: Participants were generally excited about FREED and early intervention and felt that the evidence supporting the model was compelling. As such, despite some concerns about patients outside of FREED's age criteria, the value proposition for FREED was rated as simple. Five sub-themes emerged here.

#### Sub-theme “high desirability and value”

The perceived value of FREED to patients and stakeholders was highlighted. Participants described being “ecstatic” (P2) and “excited” (P7, P12) about FREED and frequently described the model as an approach that “made sense” (P1). In some cases, participants felt that demonstrating the need for the AHSN early intervention ED programme was easier “compared to other national projects” (P12). Participants felt that FREED would have “huge potential to make a difference for young people” (P2) and have a “patient impact right from the beginning” (P9).

However, these positive and hopeful views were frequently caveated with statements highlighting concerns about operational challenges in ED services, that FREED would not be “a silver bullet” or “quick win” (P7).

*Everyone I speak to understands the … drive and the need for this. Er, which is not always the case, I'll add, in some national programmes. So, it really is refreshing. We never have naysayers as it were. We only have operational challenges.* (P9)

For the healthcare system, FREED was described as aligned with current planning to improve mental healthcare, for example, being aligned with the NHS Long Term Plan (31).

#### Sub-theme “FREED's evidence base”

The evidence base and rationale for scaling FREED were described as “robust” (P1), “compelling” (P7), “critical” (P2), and “sufficient” (P4).

…*it's been great to come across all of the peer reviewed publications about FREED … I do feel like it is very well supported and very well evidenced…* (P5)

However, participants also described some gaps in the evidence base that need to be addressed to continue building the value case for FREED. Some AHSNs had started supporting services to address these, for example, by collecting data on treatment accessibility for underrepresented and minority groups, which they felt would strengthen the programme further.

*We've also … invested in a programme to look at inequalities around eating disorders … to look at protected characteristics … we funded an evaluation of that, which we [are] just starting to reap the rewards of…* (P2)

#### Sub-theme “patients outside of the eligibility criteria”

Participants had concerns about patients who did not meet FREED eligibility criteria and desired to see FREED extended to all age groups and timely treatments delivered to all. The potential to cause “further health inequalities” (P7) sometimes caused “nervousness” (P7) among clinicians and participants, proving a challenge for getting buy-in from services.

*There is  … what feels like a slight injustice in that those who qualify or are eligible for FREED, because of … the less than three years criteria, kind of raced down the hard shoulder, as it were…* (P9)

The extra two sub-themes are given in Supplementary Material S1.

### Theme 4/NASSS domain 4: potential adopters (NHS ED clinicians) of the technology

Complexity rating: This domain was also rated as simple. Clinicians were described as having a lot of will to drive FREED forward in their service, despite some minimal hesitancy and scepticism. Two sub-themes emerged here.

#### Sub-theme “engaged and passionate clinicians”

Participants described the adopters of FREED as being engaged and passionate about early intervention in the face of operational challenges.

*The thing that hasn't been a problem, er, is the will of those people, the clinicians.* (P8)

FREED Champions were praised for advocating for early intervention and FREED and for working hard to increase motivation in their wider teams even when this was challenging.

*So in terms of will, there's lots of will. And certainly in the Champions, they will really believe in it, and they want to do it.* (P3)

There was felt to be some initial scepticism or hesitation from a few clinicians about adopting FREED. This was either due to the innovation originating from what was perceived as a well-resourced mental health trust (SLaM) or alternatively because clinicians were set in their ways. In these few cases, participants found it challenging to stir up motivation for FREED.

*That said, I'd had my fingers burnt from, er, clinical resistance and reluctance to engage with a model that was seen or could be seen to be foisted from on high.* (P11)

The second sub-theme is given in Supplementary Material S1.

### Theme 5/NASSS domain 5: the organisation (NHS ED services)

Complexity rating: The organisation was rated as complex due to various barriers around recruitment and service/leadership support. Six sub-themes emerged under this domain.

#### Sub-theme “staff recruitment and retention”

Recruitment and retention of staff were identified as significant barriers. Participants described considerable turnover and “churn” (P10) in teams. Even with flexibility in job advertisements, some services were unable to recruit into these posts, which prevented or delayed adopting FREED. This was perceived to be caused predominantly by wider recruitment issues within the NHS and pressures related to COVID-19. Many services were required to “pause” the provision of a FREED service because of staff sickness or redeployment or to prioritise high-risk cases. This was described as having “an impact on the morale” (P13) of staff. Participants described their role as “thinking creatively together” (P12) with services about how to approach recruitment issues.

*So they've had to pause it for a bit … so technically they're fully adopted because they've launched, but they have had to pause it because of the workforce issues and they're using that opportunity to look at staffing and recruitment.* (P3)

#### Leadership and internal relations of the organisation

**“**Engaged” (P12) and strong clinical leadership, with buy-in from senior clinicians and management, were described as critical to successful implementation. A lack of engagement with FREED at the management level appeared to affect implementation fidelity. For example, there were incidences where the FREED Champion's time was not protected or ringfenced for FREED activities due to a managerial focus on “the deliverables of their business as usual” (P9). This would negatively affect the capacity to complete FREED activities such as data collection.

*A concrete and fluid and, good, that's the only word I can think of, relationship between the service manager and the FREED champion is absolutely paramount to the implementation success.* (P9)

Engagement from the wider ED service was variable but important for successful implementation. In rare cases, FREED was seen as a separate entity by leadership and the wider team, which made it very difficult for the Champion to successfully implement and sustain FREED. FREED was described as working best when the Champion was supported by a mini-team and the wider team.

*We have all of the kind of suggested or required named people in place. So, we have our FREED Champions, we have the mini team … We also have … the main lead clinician who supports on the rollout as well … I think this kind of stakeholder group is really important for actually facilitating getting FREED off the ground.* (P6)

#### Sub-theme “conditions of implementation”

The rapid scaling of FREED across the country meant that some services rushed to adopt FREED before they were ready, which caused fidelity issues, for example, where Champions were working on their own rather than within a mini team. Alternatively, there were misconceptions about aspects of the model. Implementation was more successful when it took a slower pace or a step-by-step approach, focusing on building the foundations and any restructuring needed in the service. This often meant that services were more aligned to the model.

*I think some services implemented FREED potentially before they were ready to, and they hadn't done the groundwork. I think from what I'm seeing from a lot of the new services coming on board who have been working at a slower pace … their foundations are a bit stronger.* (P1)

Where the FREED Champion was working alone, participants called for more learning on the importance of a team approach to implementing FREED.

*I think the well-established sites that are confident and resilient have more of a team approach, whereas I think certainly starting up there is something of an attitude that we need the FREED Champion and that person will do everything, and that doesn't make for a resilient service*. (P13)

Within the NHS, commissioning refers to the organisations and processes involved in the planning and provision of healthcare services to meet the needs of the population, including where funding is allocated. Some ED services had only implemented FREED “partially,” such as in cases where ED services are only commissioned to treat moderate–severe EDs, a small portion of the population because of large geography, or only certain ED diagnoses. In these cases, FREED was therefore not implemented as intended. However, there was some creativity and adjustment in this, encouraged by the adoption of FREED. For example, services not commissioned to treat mild EDs made referral transitions to other provider organisations and charities.

*…they're actually only commissioned to treat moderate to severe disease. So in order to implement FREED, they actually have to then work very closely with other organizations within the trust to do - the mild [treatment].* (P3)

#### Sub-theme “balancing risks of and appetite for innovation”

Risk aversion was generally described as a barrier to the full implementation of FREED. Participants described that “whilst there was a strong desire and commitment to implement FREED, the staffing challenges … meant it was really difficult to create the space for transformation” (P9). Participants reported that, often, services would rather implement FREED on a reduced scale or delay the launch date due to worries that they “wouldn't be able to hit these targets” (P6).

… a*ctually they'd rather … squeeze the geography down or squeeze the model down to achieve the targets 100%, than take the risk … to grow the service … and just be close to those targets*. (P2)

Generally, it was acknowledged among participants that because the NHS is in a constant state of “flux” (P1) and ED services are met with tenacious operational challenges, there are inherent concerns and anxieties about making changes. However, some individual staff or services were more “innovative and open minded” (P9). Implementing FREED alongside other innovation projects or programmes helped, which meant the service was already considering transformation.

…*having an existing special service in place or launching it in collaboration with something else that's ongoing has been beneficial in getting these things launched*. (P5)

Participants were often instrumental in helping services pick a launch date for FREED to overcome some of this anxiety.

The additional two sub-themes are given in Supplementary Material S1.

### Theme 6/NASSS domain 6: the wider context

Complexity rating: This domain presented many barriers including COVID-19 disruption and differences in population and service offers across the country. However, key facilitators included excellent networking and a positive policy context for early intervention. As such, this domain was rated as complicated. Six sub-themes emerged here.

#### Sub-theme “AHSNs bringing everyone together”

AHSNs described a key element of their role as bringing all relevant groups together to facilitate the implementation of FREED. AHSNs were instrumental in forming networks and creating opportunities to share best practices among regional ED services. For example, creating meetings between ED services and commissioners was a new experience for many services:

*…I created meetings, tripartite meetings, between myself as the AHSN, the services and also commissioners. And that was fascinating because what … the service often said was ‘Oh my goodness. We've never been in a room with Commissioners before.’* (P2)

Participants created and strengthened existing pathways for communication between ED services and helped arrange meetings between clinicians and the national FREED team. Most commonly, these were community-of-practice meetings, one-to-one learning opportunities, and buddy groups for FREED Champions. These initiatives were seen as important facilitators to getting FREED started, and participants were generally satisfied that there was a “really good community around the FREED implementation*.*” (P6)

Participants highlighted the importance of embedding these initiatives so that when the AHSNs were no longer coordinating these, there would still be momentum. A potential threat to the sustainability of these was the variability of clinician engagement, confidence to share, and ability to attend these sessions due to other pressures.

#### Sub-theme “policy context for early intervention”

The wider policy context for early intervention was generally perceived as a facilitator, as many participants described FREED as aligning with the NHS Long Term Plan (31) and a focus on improving access to care for adult EDs. These policy drivers were “useful” (P3). However, participants also described challenges getting FREED on the agenda where other transformations were competing for priority funding. The transformation and “fragmentation” (P2) within the NHS were sometimes a challenge to navigate for participants in terms of ensuring FREED was included in transformation plans for mental health services.

*So there's been a lot of transformation within mental health which has helped but hindered at the same time … there's so much transformation going on, it's trying to get FREED on the agenda of, you know, the … key people.* (P1)

#### Sub-theme “geographical differences in population and service provision”

Heterogeneity within and across NHS regional areas in the accessibility of services, population density, and demographic characteristics (i.e., the proportion of young people potentially at risk of EDs) led to further complexities in the adoption of FREED. Services in rural areas often had to cover a “huge geographical area” (P7), and services in university towns had to consider that there would be “pockets of areas where there's a higher likelihood of having people who would meet the FREED criteria” (P3). Navigating these issues was felt to be challenging and sometimes led to sites “partially” adopting FREED for only some of their catchment area. Sometimes, participants thought that there was an attitude to “develop a FREED programme in certain parts of the county or area which made it kind of postcode benefit. And that's wrong” (P2).

In addition to society-wide recruitment challenges within the NHS, some participants felt that this was heightened for specific areas:

*…I think, in those areas where they already had a recruitment issue, it's sort of now really doubled or tripled.* (P13)

#### Sub-theme “COVID-19 disruption”

The COVID-19 pandemic was perceived as inevitably underlying and exacerbating many of the implementation challenges described above, but this theme describes the specific challenges brought on by the pandemic. First, participants frequently mentioned the disruption to the workforce in terms of staff sickness, turnover, and redeployment, which was perceived to impact staff morale and often shifted focus away from innovation and on to business as usual.

*Then we were seeing staff sickness, staff self-isolating. The pressure the staff were facing … you know, and their morale. It was so hard for them to manage and then trying to ask them to rethink everything er, you know it … it just wasn't an appropriate time.* (P8)

The pandemic appeared to increase “nervousness” (P1) about adopting FREED and about the potential of “launching a service and being absolutely inundated. Due to the numbers” (P2), some services had to pause FREED for pandemic-related reasons but were able to restart FREED when they had the capacity. In some cases, participants' abilities to connect with people to get FREED started were affected, as opportunities to meet “face to face … to connect” (P10) were restricted.

Two additional sub-themes are provided in Supplementary Material S1.

### Theme 7/NASSS domain 7: sustainability and emergence over time

This domain was rated as complicated; whilst there were some concerns and uncertainty about the future of FREED, participants mostly remained positive and optimistic. Three sub-themes emerged here.

#### Sub-theme “need for flexibility and creativity”

When discussing the sustainability of FREED, participants often expressed that this will be contingent on allowing flexibility or creativity within the pathway, for example, with data collection requirements (P7), the FREED Champion post (P12), and what options are available for treatment under the FREED pathway (P5). Two participants discussed the potential of having core FREED principles and values but allowing waiting time targets or treatment options to be more flexible:

*It would be nice to sort of have really clear, like … what's the clear … aim of FREED in terms of those … core values, but with some … options around how you could deliver it or what's allowed. What's allowed that makes it still FREED versus what's like, actually now you’re doing something completely different.* (P3)

Some also mentioned how this impacted patient care, for example, by having “had some success with doing … active support groups virtually” (P3).

#### Sub-theme “implementation and network sustainability”

Generally, participants had positive views on the sustainability of FREED, describing the pathway as “adaptable and scalable” (P4) and “a sustainable model” (P2). These views were frequently tempered with concerns about implementation fidelity and funding stability. For example, participants wanted to ensure that “the fidelity is maintained, to ensure those outcomes are maintained” (P1).

Participants were concerned about newly launched services that may require support beyond the end of the national programme, and without the focus of a national programme, momentum could “wither on the vine” (P10). Some participants were helping sites to create sustainability plans regarding funding for the future beyond AHSN support (P2, P3).

Participants remained positive and felt that there was “real commitment from the ground for this” and that “the will is there” (P9) to make FREED work despite some of the challenges and concerns around sustainability.

*I do think it's sustainable … I don't think we have a choice for it to be sustainable or not. We can't. Early intervention isn't an option, it's a necessity*. (P9)

An additional sub-theme is given in Supplementary Material S1.

## Discussion

This study aimed to explore the perceived facilitators and challenges associated with implementing FREED in ED services across England and to seek views on the sustainability of the model from the unique perspective of AHSN implementation specialists. The application of the NASSS framework supported the categorisation of implementation facilitators and barriers and areas where complexity is present across each interconnecting domain. Prominent facilitators were a high-value proposition of FREED and engaged, passionate adopters. AHSN participants were largely supportive of early intervention and could see the potential for positive patient outcomes. The complexity analysis suggests the greatest challenges to implementation are illness-related factors and organisational barriers. As the national AHSN programme has ended, complexity may endure and/or increase, and strategies to manage this in the sustainability phase of FREED will need to be monitored. When thinking towards the future, participants expressed concerns about the stability of implementation and networking across services using FREED and stressed the importance of flexibility and creativity in local adaptations of FREED.

Even when coping with circumstances such as the COVID-19 pandemic and an increase in patient referrals, AHSN leads felt that there was a will among clinicians in ED services to adopt FREED. Further, FREED Champions worked hard to implement FREED and increase enthusiasm in their wider teams. How adopters perceive a healthcare innovation and their beliefs and values about patient care are essential to innovation engagement and adherence (22, 32). Innovations that go against the intended users' values or that they cannot see advantages in are less likely to be supported (21, 33). The role of FREED Champions appeared to be an integral part of the perceived success of FREED, and AHSN participants discussed the importance of sustaining the funding for these posts to ensure that early intervention is continually championed across ED services. Ensuring protected spaces for clinicians to share learning and best practices was also important to participants. As some services are still early in their implementation journey, ensuring they have these forums to adopt FREED with optimal fidelity will be important. Careful planning of how the FREED network will operate after the AHSN EIED programme ends is therefore required.

The perceived value of an innovation is arguably one of the most important enabling factors for scaling, spreading, and sustaining a healthcare innovation (34). AHSN leads believed that FREED could demonstrate high value for patients and that FREED's evidence base was sufficient and compelling. For example, FREED has demonstrated reductions in day/inpatient admissions, which translates to considerable cost savings for the NHS (9). Therefore, we considered the value proposition for FREED to be simple. However, participants desired to see additional research on treatment accessibility for underrepresented groups and further evaluation of the national scaling of FREED. Ultimately, more robust evidence, such as that provided via randomised controlled trials evaluating FREED against credible control interventions, is needed. Early intervention initiatives for those over 25 years are lacking (6), and whilst ethnic minority and LGBTQIA+ people may be more likely to experience EDs (35–37), they may be underrepresented in ED services (38). Research addressing some of these gaps is currently in progress (www.EDIFYresearch.co.uk) (39).

The condition domain was determined to be complex (unpredictable, multiple interacting components) when taken together with what we know about EDs. EDs are highly varied and comorbid with other psychiatric and physical health conditions and carry long-term morbidity and mortality burdens for the individual (40, 41). Furthermore, EDs are often stigmatised, under-researched (7), and disproportionately affect certain groups of people, as has been discussed. Participants frequently expressed concerns about services' abilities to cope with an increase in the number and acuity of ED referrals observed since the onset of the COVID-19 pandemic. Our own data show that FREED services experienced a ∼1.4-fold increase in referrals after the onset of the pandemic, which calls for careful continued monitoring of the long-term impacts of the pandemic (19). Participants also considered issues around help-seeking in EDs as a potential barrier to early intervention. The FREED team has developed a mobile application (FREED-M), which is currently being evaluated in a feasibility trial assessing whether FREED-M can improve help-seeking in young people with an ED (42). Other ways to manage complexities related to EDs must be considered.

One of the main factors presenting as a facilitator or barrier under the technology theme was related to funding options; participants desired more investment opportunities for ED services and described a lack of continued funding for FREED as a source of concern. When discussing sustainability and “futureproofing” FREED, most participants highlighted the need for continued investment, which aligns with recommendations to invest in ED services and early intervention initiatives to cope with the increased demand (6, 20). Complexity emerged as the implementation of FREED requires clinicians to deliver NICE-recommended treatments, and participants stated that more training opportunities to upskill clinicians to deliver these are needed. There was also a desire to see training on managing age-related transitions of care in a FREED context. Such a training package is currently being developed.

The organisation was rated as complex, where barriers included variable leadership/team support for FREED. Sufficient clinical leadership and support are central to implementation projects and innovation in healthcare, and leaders are essential in cultivating an environment where staff feel confident to take appropriate risks and engage in innovation (43). However, adult ED services have historically been under-resourced, and the NHS has an ethos of gatekeeping to optimise scarce resources (11, 20), which, as described by some participants, has led to anxiety about change and risk aversion. The AHSNs were instrumental in helping address risk aversion by ensuring sites could implement gradually and by encouraging teams to decide launch dates. The support of the AHSN has therefore undoubtedly facilitated and encouraged services to launch FREED. However, other crucial challenges across the NHS include significant staff recruitment difficulties and unfilled vacancies (44). Participants identified struggles to recruit and keep FREED Champions, which could pose a threat to the sustainability of FREED. Also, as FREED currently operates on a “train the trainer” approach, a high turnover of staff could lead to a lack of consistent messaging about the principles of the FREED pathway. These accounts of the impact of recruitment challenges highlight the need for assessments of implementation fidelity to ensure consistent messaging and practice. Adopting a team-based approach to FREED may also help guard against deteriorating fidelity as one person does not have to solely uphold the pathway and values.

Important issues concerning the sustainability of FREED over time included, first, implementation fragility, for example, a “partial” adoption of FREED, or “pausing” of FREED. Participants highlighted a need for flexibility when implementing the pathway, allowing at least to start with a partial adoption (e.g., within part of a service's catchment area or covering only certain diagnostic groups). Greenhalgh and Papoutsi (43) stress that individuals create adaptive solutions according to their local contexts, creating an inevitable gap between the evidence-based ideal and the real-world context, but that these workarounds and amendments should be studied carefully. These workarounds appeared to help stretched services adopt FREED at a slower pace. Nonetheless, as FREED has now been adopted across an increasing number of ED services and organisations, it is important to investigate whether FREED is being delivered as intended, i.e., as a transdiagnostic service model covering all ED diagnoses and severities. Assessments of implementation fidelity should allow for some flexibility in local adaptations, and it appears these have been an important factor for the realistic adoption of FREED, but it should also be determined how much and which adaptations are acceptable and which are not (45). The latter is important to avoid a delivery of “watered down” early intervention and FREED “in name only.” For example, if FREED is only being rolled out for certain diagnoses (i.e., anorexia nervosa and other low-weight presentations), this will disadvantage young people with bulimic presentations at higher weight, a group that is often underserved and experiences multiple systemic and health disadvantages (46).

### Strengths and limitations

The strengths of this study include gaining a rich understanding of FREED implementation barriers and facilitators from stakeholders external to the FREED national team, who are experts in scaling healthcare innovations. Therefore, participants were able to draw on their unique perspective from scaling other health innovation projects in the NHS. Additionally, most AHSN regions participated in this study, meaning views representing most NHS regions across England were included. A potential limitation is that there may have been a degree of bias: AHSN leads work to promote FREED to stakeholders and services and may have been partial to favourable statements about FREED and a tendency to focus on the benefits of the programme. However, the interview topic guide encouraged them to draw comparisons between their work on FREED and that in other implementation projects and strongly encouraged confidential expression of negative and positive views.

## Conclusion

This study revealed several sources of possible complexity relevant to implementing the FREED pathway across ED services in England. However, central to most of the interviews were descriptions of services, clinicians, and stakeholders being enthusiastic about early intervention and FREED. In recent years, clinical, research, and campaign groups have strongly advocated for investment in early intervention for EDs, setting a strong policy consensus for early intervention (6, 12, 13, 47). The experiences of FREED patients and other individuals with lived experience also confirm that early access to treatment is a priority (6, 16). Despite operational complexities, we believe that this momentum and support will help drive FREED forward. Collaborating with the AHSNs has been of great benefit for scaling FREED across the NHS, and these organisations have been identified as a key component to creating an innovative work culture in the NHS (48). Work must focus on sustaining investment, improving fidelity, and futureproofing FREED beyond the national programme.

## Data Availability

The datasets presented in this article are not readily available because participants did not give explicit written consent for their data to be publicly shared. Upon reasonable request, the processed dataset can be supplied by the corresponding author. Requests to access the datasets should be directed to lucy.e.hyam@kcl.ac.uk.
