# Supplementary material for: “Early intervention isn't an option, it's a necessity”: learning from implementation facilitators and challenges from the rapid scaling of an early intervention eating disorders programme in England
Source: Front Health Serv. 2024 Jan 18;3:1253966. doi: 10.3389/frhs.2023.1253966 (PMC10830832; doi:10.3389/frhs.2023.1253966)
Supplement: Supplementary file 1 [file Datasheet1.pdf]

Supplementary material 1: Additional sub-theme data

Theme 1/NASSS Domain 1: The condition

Sub-theme ‘Understanding of eating disorders’

Many participants expressed having a good understanding of EDs and some had clinical experience working in this area. Those that didn’t, quickly engaged with the FREED evidence base and other resources.

> *“…one of the…first things I learned about the programme was around this like, biological malleability model where the younger you are the more, essentially, amenable you are to change or intervention.”* (P6)

Participants often mentioned the general lack of understanding more widely in healthcare/society, that EDs are “*not well looked after, not well funded, and in general not well or not fully researched*” (P5), and that there is “*a lot of taboo around [EDs]*" (P10).

However, some participants felt that public understanding/interest in EDs was increasing due to a rise in media coverage on ED referrals during the COVID-19 pandemic, that “*the predominance of eating disorders within the community, that was being made evident from the pandemic, hit national press.*” (P4) Participants’ understanding of EDs and the need for early intervention appeared to kindle their own enthusiasm for FREED.

Theme 2/NASSS Domain 2: The technology (FREED)

Sub-theme ‘Adhering to waiting time targets’

Key features of the FREED pathway are the waiting time targets for assessment and treatment. Some services were mostly adhering to the targets. In these cases, participants described the targets as “*sustainable*” (P5). Where sites were struggling to meet targets due to capacity concerns, participants described the waiting time targets as sustainable “*in theory*” (P11), given the right investment into FREED and the workforce.

Overall, participants recognised that wait time problems arose because of external factors (e.g., increased referrals) as opposed to problems with the targets themselves.

> “*I think that they are sustainable, so I wouldn't say ‘Oh no, it's not possible.’ I think that we're just gonna have to put in a lot of work in order to get to those waiting time targets.*” (P11)

Theme 3/NASSS Domain 3: The value proposition

Participants were generally excited about FREED and early intervention and felt that the evidence supporting the model was compelling. As such, despite some concerns about patients outside of FREED’s age criteria, the value proposition for FREED was rated as simple.

Sub-theme: ‘Capturing the wider benefits of FREED’

A barrier described, particularly when discussing making a business case for FREED, was that the AHSN programme measures of value did not necessarily capture the whole picture of FREED

benefits. Participants described an organisational "*fixation*" (P2) on adherence to the waiting time targets and numbers of patients receiving evidence-based treatment in the FREED pathway as the main sources of value generation. However, programme leads desired recognition of the wider range of benefits and sources of value generated from adopting and implementing FREED, including service redesign and the "*work to keep in contact with them [patients], to give them some support while they're waiting for their evidence-based treatments.*" (P1).

> *"I think there's a huge amount of benefit, which comes along which goes far beyond the identified outputs and outcomes of the FREED national programme."* (P4)

Sub-theme: 'Buy-in required across multiple systems'

Obtaining buy-in across multiple systems was often described as a necessity to getting FREED adopted, but was often a challenge. Engagement was required from NHS England, integrated care systems (ICSs), clinical commissioning groups (CCGs), senior leadership teams and clinicians, and sometimes other treatment provider organisations. Integrated care systems (ICSs) are regional partnership organisations across England responsible for planning and funding healthcare services that meet the needs of local areas. They replaced clinical commissioning groups from July 2022, which held the same responsibility prior to being dissolved(1).

Buy-in across these multiple systems was difficult for participants, who noted that "*trying to pin down the right people to move forward with can sometimes prove quite a difficulty.*" (P13).

> "*…getting that buy-in across complex systems. That's a real challenge to getting started*." (P3)

However, some participants found this easier due to their existing connections across these systems.

Theme 4/NASSS Domain 4: Potential adopters (NHS ED clinicians) of the technology

Sub-theme: 'Support needed from AHSN'

The AHSN leads perceived their support as integral to implementation. Levels of support varied according to implementation progress, with some services requiring lots of "*hand-holding*" (P7) and others only requiring "*light touch*" (P3) support. AHSN leads provided programme management support tailored to local needs, ensured FREED training had been completed, monitored fidelity to the FREED model, and helped with FREED data collection issues. One AHSN had developed a data visualisation tool to help services assess local impact. Programme management needs were sometimes perceived as greater than those required in other national programmes, due to the operational challenges experienced in ED services.

"*So, with some national programmes we can sit outside of the project delivery side of it to a degree. And with this programme, I really took a hands-on approach...*" (P7)

Theme 5/NASSS Domain 5: The organisation (NHS ED services; Domain 5)

Sub-theme: ‘Limited capacity of ED services’

ED services were described as stretched to their capacity limit, as such, FREED is “…*a difficult ask of the services because they're so busy and already overstretched*” (P9). Challenges included coping with an increase in referrals, long wait times for treatment, and managing these alongside the pressures of the COVID-19 pandemic. In some cases, this meant that FREED was not working to its full potential, “*because early intervention is meant to…speed things up and…it hasn't really*”. (P11)

AHSN leads also felt that clinicians often had difficulties collecting data as part of FREED. Whilst participants noted that data collection requirements were as “*minimal as possible*” (P5), clinicians struggled to find the time or resource to “*manually input a lot of…data*” (P2) and there were sometimes issues with data quality.

Sub-theme: ‘Fit between ED services and FREED’

Overall, FREED was described as a flexible, adaptable pathway with a good fit to ED services; it was felt that FREED was “*pragmatic in its approach*” (P10). Many participants described the design of FREED as more of a “*mindset shift*” (P4) and “*a tweak to existing pathways and to existing ways of working*” (P13) as opposed to a launch of a whole new service.

> “*I think what it's really doing is encouraging our services to think about how the FREED pathway would fit into that already occurring service… You're already doing this, it's just thinking about how we can slightly change our efficiency or our staffing to be able to hit some of those much shorter… referral and treatment time points.*” (P6)

Whilst FREED was generally described as a good fit to ED services, it was acknowledged that some services required more planning and support to implement FREED than others. For example, having an all-age ED service was perceived as “*a real benefit*” (P2) due to FREED’s target age range of 16–25-year-olds. Conversely, where ED services were split between child and adolescent (up to 16/18 years old) and adult (16/18+ years old) ED services, this was reported as “*very difficult*” (P13). Most expressed a desire for more support, research, and training into how to best manage transitions of care when implementing a FREED service.

Theme 6/NASSS Domain 6: The wider context

Sub-theme: ‘Relationships between AHSN, adopters, and KCL/SLaM FREED national team’

Both the relationships between AHSNs and the FREED national team, and the relationship between AHSNs and adopters were described as facilitators to implementing FREED. AHSNs felt supported by the national team and appreciated their openness to communication. As such, AHSNs were able to feedback local issues:

> “*I think the support from the FREED Network has been amazing and I think without working in such close partnership with [the FREED national team] that it would have been impossible for us to… scale FREED as we have*.” (P1)

AHSNs worked hard to build trust and relationships with FREED clinicians and ED services. Many participants reported that clinicians openly came to them for advice and that there was "*good dialogue and open communication*" (P6). Participants worked hard to understand local issues and barriers relevant to FREED implementation to best support and "*be champions for*" (P10) services.

Sub-theme: 'Good engagement with patient and carer groups and charities'

Participants highlighted a good relationship with university health and wellbeing services, patient communities, and voluntary/charity organisations as a facilitator to increasing stakeholder buy-in for FREED. When these groups were able to 'back' FREED and the need for early intervention for their area, it built momentum and "*kickstarted the service*" (P11).

> "*The inclusion of the patient and carer collaborative has been a wonderful driving force… it has, more than any other programme I'm working on, the feeling of a cause as much as an implementation.*" (P9)

Where patient involvement groups were not part of mental health trusts, it was more difficult to get FREED noted by executives. Participants desired to see these kinds of relationships grow and for university health and wellbeing services and FREED teams particularly to increase networking.

Theme 7/NASSS Domain 7: Sustainability and emergence over time

Sub-theme: 'Increasing referrals and condition complexity'

Participants considered how the condition may change over time and wondered whether the "*astronomical"* (P9) increase in referrals witnessed at the time of interview would continue. Additionally, there was a perceived increase in comorbidities and concerns about "*severe*" (P1) and "*more complex*" (P12) referrals coming through due to COVID-19. This was generally perceived as a source of pessimism for participants when thinking about the future of FREED.

References

1. NHS Confederation (2022). Integrated care systems (ICS): parliamentary briefing. https://www.nhsconfed.org/publications/integrated-care-systems-ics [Accessed 09 May, 2023].
